# Evaluation of the adaptogenic potential exerted by ginsenosides Rb1 and Rg1 against oxidative stress-mediated neurotoxicity in an *in vitro* neuronal model

**DOI:** 10.1371/journal.pone.0182933

**Published:** 2017-08-16

**Authors:** Carlos Fernández-Moriano, Elena González-Burgos, Irene Iglesias, Rafael Lozano, M. Pilar Gómez-Serranillos

**Affiliations:** 1 Department of Pharmacology, School of Pharmacy, University Complutense of Madrid, Madrid, Spain; 2 Department of Inorganic Chemistry, School of Pharmacy, University Complutense of Madrid, Madrid, Spain; The University of Texas at El Paso, UNITED STATES

## Abstract

**Background:**

Ginseng (*Panax* sp.) is a drug with multiple pharmacological actions that has been largely used in traditional medicines for the treatment of many health problems. In the therapy of neurodegenerative disorders, it has been employed due to its capacity to strengthen mental processes by enhancing cognitive performance and psychological function. Current work aimed at evaluating the adaptogenic potential of Rb1 and Rg1 against oxidative-stress mediated degeneration in a model of nervous cells.

**Methods:**

Oxidative stress and mitochondrial dysfunction were achieved by exposing SH-SY5Y cells to the mitochondrial complex I inhibitor rotenone. The cytoprotective activity of pre-treatments with ginsenosides Rb1 and Rg1 against rotenone was assessed by determining biochemical markers regarding oxidative stress (ROS scavenging, glutathione and lipid peroxidation levels, SOD activity and Nrf2 activation) and apoptosis-related alterations (mitochondrial membrane potential, calcium levels, aconitase activity and pro/antiapoptotic proteins). Their capacity to cross the blood brain barrier was also estimated.

**Results:**

At their optimal doses, ginsenosides Rb1 and Rg1 significantly ameliorated redox status within the cells; they reduced ROS and TBARS levels and improved the glutathione system, as well as they enhanced SOD activity and Nrf2 pathway activation. They protected neuronal cells against MMP loss, calcium homeostasis disruption and aconitase inhibition. Consequently, apoptotic cell death was attenuated by the pre-treatment with ginsenosides, as evidenced by the reduction in caspase-3 and Bax, and the increase in Bcl-2 expressions; also, lower levels of cytochrome C were found in the cytosol. Poor BBB permeation was demonstrated for both ginsenosides.

**Conclusions:**

In conclusion, ginsenosides Rb1 and Rg1 exhibit neuroprotective potential which is achieved, at least in part, via mitochondrial protection and the plausible involvement of Nrf2 pathway activation. Our results contribute to validate the traditional use of ginseng for cognitive-enhancing purposes and provide basis to encourage further research on the potential of ginsenosides in the treatment of neurodegenerative diseases.

## Introduction

Chronic age-related neurodegenerative disorders suppose a worldwide leading cause of death and disability, especially in the elderly over the age of 60, and involve an incredibly high economic cost; for instance, World Health Organization (WHO) estimated in 2015 that over 47 million people suffered from Alzheimer´s-like dementia and this prevalence is supposed to increase in the near future. Consistent evidences support the idea that neurodegenerative diseases (such as Parkinson´s and Alzheimer´s diseases) are directly linked to a harmful situation of cellular oxidative stress within the central nervous system (CNS) [1]. It is not completely clear whether oxidative stress is a causative or consequential factor in age-related neurodegeneration, but the imbalance in pro-oxidant/antioxidant homeostasis is acknowledged to occur in the brain of patients. An eventual over-production of toxic reactive oxygen species (ROS) affects most of the cellular biomolecules, such as DNA, membrane lipids or active proteins [2]. The failure in physiological adaptation against the noxious environment leads to subsequent mitochondrial dysfunction, abnormal protein folding and aggregation and metal ion imbalances, among other contributing events, that provoke the degeneration of nervous cells [3]. The multifactorial etiology of these diseases and the lack of effective diagnosis methods imply the scarce effectiveness of the available treatments, which are mainly symptomatic once the neuronal damage is irreversible. Therefore, there is an urgent need for novel neuroprotective therapies that reduce mortality and prevent or delay the onset of symptoms.

Traditional medicines, and especially traditional Chinese medicine (TCM), has largely dealt with the treatment of aging and related neurodegenerative disorders, usually having good clinical tolerability as drugs used are mainly from natural origin with fewer side effects. Among all herbal preparations used with this aim, ginseng certainly deserves to be highlighted [4].

‘Ginseng’ is the popular name taken to designate the drug consisting of the dried root of several species that belong to the plant genus *Panax* (Araliaceae family). Although the most used species is *Panax ginseng* C.A. Meyer (the one growing most notably in China and Korea), it also refers to other members of the genus, including *Panax quinquefolius* (American ginseng), *Panax notoginseng* and *Panax japonicus*. It has a medical history longer than 5000 years and has been generally employed in the Far East as a panacea due to its restorative and tonic functions; but its medicinal use has been properly explored not only in East Asia, but also in the Soviet Union and the USA [5]. Newall and coworkers [6] highlighted the traditional use of ginseng as a long-term drug to favor the recovery of unhealthy individuals and in age-related degenerative conditions. As a tonic drug, it favors the resistance to fatigue and various stressors and it is effective against cerebral hypoxia and alterations in neurotransmitters [7]. Based on TCM and modern pharmacological theories, ginseng is currently one of the most widely taken herbal products throughout the world and has a high value and potential not only as a general tonic but also in the therapy of neurodegenerative disorders due to its capacity to strengthen mental processes, by enhancing cognitive performance and psychological function [8]. The capability of ginseng to counteract cellular oxidative stress and uphold homeostasis may mediate its neuroprotective effects. Actually, it has recently been reported that ginseng extracts show cognition-enhancing effects in Alzheimer´s disease (AD) patients [9] and in diverse models of Parkinson´s disease (PD) [10]. The protective activity of ginseng on neurodegenerative disorders has been reviewed by Ong and coworkers [11].

The most relevant active principles present in ginseng root and responsible for its biological activities are triterpene saponins known as ginsenosides. Among them, the dammarane-type ginsenosides Rb1 and Rg1 are two of the most abundant and active compounds from a pharmacological point of view. These isolated compounds have been proved to exert diverse effects, as it is evidenced by their potential against many health problems such as liver fibrosis [12], physical stress [13] and even cancer [14], among others. What is more, they have shown effectiveness in conditions affecting CNS, such as spinal cord injury [15] and cerebral ischemia [16]. The interest of ginsenosides Rb1 and Rg1 in the treatment of age-related neurodegenerative diseases mainly relies on their cytoprotective effects against oxidative stress-mediated apoptosis and other neuronal disturbances [17]. For instance, Rb1 and Rg1 demonstrated *in vivo* anti-amnestic and anti-aging effects via restoration of redox homeostasis and inhibition of neuronal apoptosis [18]; also, they ameliorated cognition-deficiency in mice with dementia, with enhancement of acetylcholine in hippocampus [19] Regarding PD models, Rg1 displayed *in vivo* protection against MPTP-induced apoptosis in the *substantia nigra* [20], and both Rb1 and Rg1 preserved structure and function of dopaminergic neurons from MPP+ damage due to their antioxidant properties [21]. These and other studies provide solid basis to encourage a deeper study of the mechanism of their neuroprotective actions.

In the recent years, the use of exogenous rotenone in *in vitro* models (mostly CNS-derived primary cultures or cell lines) has been extensively adopted as a model of oxidative stress and mitochondrial dysfunction for studies of neuroprotection, as rotenone selectively inhibits mitochondrial complex I [22]. When used in dopaminergic cells, as it is the case of the human neuroblastoma SH-SY5Y cell line [23], it is accepted to mimic an *in vitro* condition closely related to the neurodegeneration occurring in the *substantia nigra* of PD patients and it is commonly used for the study of neuroprotective strategies [24]. To our knowledge, no previous studies have evaluated the effects displayed by ginsenosides Rb1 and Rg1 in such model.

Therefore, in view of this information, the current work aimed at evaluating for the first time the adaptogenic and cytoprotective potential of ginsenosides Rb1 and Rg1 against the oxidative stress, mitochondrial dysfunction and apoptosis mediated by rotenone in the SH-SY5Y cell model. Herein, we further study their possible mechanism of action as well as their capacity to cross BBB, as a necessary requirement for them to display their neuroprotective actions in the brain.

## Material and methods

### Reagents

Most of the chemicals used in the experiments were obtained Sigma-Aldrich (St Louis, MO), such as 2,2´-azobis (2-methylpropionamidine) dihydrochloride (AAPH), 2,7-dichlorofluorescein diacetate (DCFH-DA), 3-(4,5-dimethylthiazol- 2-yl)-2,5-diphenyl-tetrazolium bromide (MTT), rotenone, sodium dodecyl sulfate (SDS), Triton X-100 and Trolox, as well as the anti-ß-actin (A2228), anti-cytochrome C (SAB4502234) and the anti-mouse secondary (A9044) antibodies. Other antibodies for Western blot assay were from different suppliers: anti-Nrf2 (sc-365949), TFIIB (sc- 271784), goat anti-rabbit (sc-2004 and sc-516102) from Santa Cruz Biotechnology (Santa Cruz, CA, USA), and caspase-3 (A2156), Bax (A0207) and Bcl-2 (A0208) from NeoBiolab (UK). Dulbecco’s modified Eagle’s medium (DMEM), fetal bovine serum (FBS), trypsin-EDTA, phosphate buffer saline (PBS), Rhod-2-AM, Indo-1/AM, and calcium ionophore A23187 were obtained from Invitrogen (Carlsbad, CA, USA). Panreac (Barcelona, Spain) supplied the dimethyl sulphoxide (DMSO), and methanol and acetonitrile of HPLC-grade. The SH-SY5Y cell line was provided by the University of Alcalá (Madrid, Spain), CAI Medicine and Biology, Cell Culture Unity.

### Chemical compounds

Pure ginsenosides Rb1 (Ref. 0105S, batch: 34; PubChem CID: 9898279), Rg1 (Ref. 0101S, batch: 05092030; PubChem CID: 441923), Rb2 (Ref. 0104S, batch: 05092019), Rc (Ref. 0106S, batch: 03051614), Rd (Ref. 0102S, batch: 050992012) and Re (Ref. 0103S, batch: 10) were purchased from Extrasynthese (Genay, France) with a degree of purity of ≥ 98%, ≥ 97%, ≥ 97%, ≥ 98%, ≥98% and ≥ 98%, respectively. Stock solutions of individual ginsenosides (500 μM) were prepared in DMSO and diluted in PBS before use.

### Radical scavenging capacities

The oxygen radical antioxidant capacity (ORAC) assay was performed following the method previously described [25]. In brief, trolox (water-soluble vitamin E analog, used as reference antioxidant) and ginsenosides were progressively diluted in PBS from a methanol stock solution of 1 mg/mL. Different concentrations of ginsenosides were incubated in 96-well opaque plates with fluorescein (70 nM) for 10 min at 37°C. The subsequent addition of AAPH (12 mM) initiated the reaction of peroxyl radicals generation through its thermal decomposition. Fluorescence was then recorded for 98 min at λ_exc_ 485 nm and λ_em_ 520 nm in a FLUOstar OPTIMA fluorimeter (BMG Labtech, Ortenberg, Germany). ORAC results are expressed as μmol Trolox equivalents (TE)/mg sample, obtained from the comparison of the area under the curve (AUC) of each sample with that of Trolox.

### SH-SY5Y cell line: Culture and treatments

SH-SY5Y cells [26] were maintained in DMEM supplemented with 10% FBS and 0.5% gentamicin (50 mg/mL) at 37°C in a humidified atmosphere with 5% CO_2_/95% air, and then seeded in 96 and 24-well plates and in 100-mm culture plates for the different experiments. Cells were sub-cultured until a maximum passage number of 10.

For cell treatments, SH-SY5Y cells were pre-incubated for 24 h with various doses of ginsenosides before rotenone 50 μM was added for other 24h [27]. Rotenone was first dissolved in DMSO and then diluted in PBS. In all cases, final DMSO concentration was lower than 0.1% in the plates.

### Assessment of cell viability

The MTT reduction assay [28] was carried out as an indicator of cell viability and mitochondrial function. After treatments, medium was removed and cell viability was measured by incubating cells with 100 μL of MTT (2 mg/mL) for 1 h and at 37°C. Formazan blue crystals generated by living cells were dissolved in DMSO and the absorbance was measured at 550 nm using a Spectrostar Nanomicroplate reader (BMG Labtech, Ortenberg, Germany). Results are expressed as the percentage of viable cells in comparison to control cells (taken as 100% absorbance). MTT assay was used for assessing the effects on cell viability of several concentrations of ginsenosides (by themselves), as well as for the evaluation of their cytoprotection against rotenone (pre-treatments with ginsenosides and later addition of rotenone).

Cellular morphology was examined after the different treatments by phase contrast microscopy and photographs were taken using a Motic Moticam 2500 camera.

### Measurement of oxidative stress markers

#### Glutathione system

The levels of the reduced active form of the antioxidant glutathione (GSH) and its oxidized form were titrated by the method of Hissin and Hilf [29], based on the reaction with o-phthaldehyde (OPT) and the measurement of subsequent fluorescence. Procedure was fully reported by González-Burgos and coworkers [30]. In brief, we prepared total cellular extracts. After treatments, cells were collected and homogenized in a phosphate buffer containing 0.1 M NaH_2_PO_4_ and Na_2_HPO_4_, and 5 mM EDTA (pH 8). Cells were then sonicated (10 s, 100% amplitude) in ice for favoring membrane lysis. Homogenates were centrifuges (2500 rpm, 10 min, 4°C) and glutathione was subsequently determined in the supernatans (total extracts), where proteins were also titrated by the bicinchoninic acid method. In addition, proteins were precipitated with perchloric acid 1% (1.5 μl/ml) before fluorescence measurements.

#### Intracellular ROS generation

ROS production was evaluated by the DCFH-DA method [31], with minor modifications. Briefly, after cells treatments in 96-well plate, 200 μL of a 10 μM solution of DCFH-DA in PBS-glucose were added for 30 min and incubated at 37°C. Then, cells were washed twice with PBS-glucose and ROS generation at the intracellular level was examined for 2 h at λ_exc_ 480 nm and λ_em_ 510 nm in a microplate fluorescence reader (FLx800, Bio-Tek Instruments INC, Germany). Results are given in % of ROS generation in comparison to control cells at the end of the experiment.

#### Thiobarbituric acid reactive substances (TBARS) levels

Lipid peroxidation in cell membranes was estimated by the method of Mihara and Uchiyama [32], which measures the formation of TBARS. Briefly, 50 μL of total extracts (firstly frozen at -80°C and defrost at room temperature), were mixed with 100 μL of TBA-TCA-HCl reaction mixture, and samples were then boiled at 100°C for 10 min. Reaction was stopped on ice and samples were centrifuged at 3000 rpm for 10 min at 4°C. Finally, the absorbance was read at 535 nm using a Spectrostar Nanomicroplate reader.

#### Superoxide dismutase (SOD) enzyme activity assay

SOD activity was measured following a method previously described [33] with some modifications. Total cell lysates (25 μL) were added to a reaction mixture (final volume of 800 μL) composed of 50 mM Tris-DTPA buffer (pH 8.2) and 15 μL of pyrogallol diluted into 10 mM HCl; absorbance was then determined at 420 nm for 1 min.

### Evaluation of mitochondrial dysfunction and related apoptosis

#### Mitochondrial membrane potential (ΔΨm)

We used the fluorescent cationic dye tetramethylrhodamine methyl ester (TMRM) with the aim to monitor the mitochondrial membrane potential. After treatments, cells were incubated in Krebs medium containing TMRM 150 nM and fluorescence intensity was measured at λ_exc_ 549 nm and λ_em_ 573 nm during 45 min at 37°C using a FLUOstar Optima. Then, FCCP (6 μM) and oligomycin (0.25 μg/mL) were added for uncoupling mitochondrial potential, what resulted in maximum depolarization. Next, fluorescence intensity was measured under identical conditions for 15 mins. Calculations of ΔΨm were obtained by subtracting the maximum fluorescence (values obtained after FCCP and oligomycin) and basal fluorescence values.

#### Cytosolic and mitochondrial calcium levels

The determination of mitochondrial calcium uptake was achieved by the use of the fluorescent cationic dye Rhod-2/AM, while the cytosolic levels of calcium were titrated through the utilization of the specific dye Indo-1/AM. Specific details on the methods and calculations are collected in a previous publication of our group [34].

For the determination of mitochondrial calcium, 2×10^5^ cells were seeded in 24-well plates and incubated for 24 h to reach cellular confluence. After treatments, cells were incubated with the fluorescent dye (for the hydrolysis of its ester form) and fluorescence intensity was measured at λ_exc_ 552 nm and λ_em_ 581 nm for 5 min. Then, the calcium ionophore A23187 (5 μM) was added and fluorescence was again recorded for 15 min under the same conditions. The difference between the maximum fluorescence intensity due to ionophore addition and basal fluorescence was used for determining mitochondrial calcium uptake. Results are expressed normalized to control cells (basal Ca^2+^ content in mitochondria).

The quantification of cytosolic calcium levels is addressed through the incubation of cells, after treatments, with the fluorescent dye Indo-1/AM 3 μM in Krebs Medium (45 min, 37°C, and darkness). Cells are subsequently maintained in dye-free medium (15 min, 37°C) to favor ester hydrolysis, and fluorescence is measured for 4 min at λ_exc_ 350 nm and λ_em_ 410 nm (37°C), in a FLUOstar OPTIMA. Then, ionomycin (3 μM) is added to the wells and fluorescence intensity is again recorded for 8 min under the same conditions. Finally, with the addition of MnCl_2_ (3 mM), the last measurement of fluorescence lasts for 4 min. Cytosolic calcium levels are calculated by using the following formula: [Ca^2+^]i = K_d_ × (F − F_min_)/(F_max_ − F); where K_d_ = dissociation constant of Indo-1 (250 nM), F = relative fluorescence signal of samples, F_min_ = AF + 1/12 (F_max_ − AF), F_max_ = maximum fluorescence signal after ionomycin, and AF = auto-fluorescence signal obtained after MnCl_2_ addition.

#### Caspase -3 activity assay

Inhibition of rotenone-mediated apoptosis by ginsenosides was estimated by measuring the activity of caspase-3 enzyme through the cleavage of the fluorogenic substrate Ac-DEVD-AMC. SH-SY5Y cells were seeded in 24-well plates (3×10^5^ cells/well) and, after 24 h, cells were treated and then lysed. Cell lysates were centrifuged at 13,000 g for 5 min and the supernatants (with at least 20 μg) were used for enzymatic measurements; they were incubated at 37°C (1 to 6 hours) in caspase-3 assay buffer (20 mM, HEPES, 2 mM DTT, pH 7.5) and 20 mM Ac-DEVD-AMC. Fluorescence intensity was measured at λ_exc_ 360/40 nm and λ_em_ 460/20 nm using a microplate fluorescence reader. Results are expressed as percentage of basal activity in control cells (100%).

#### Aconitase activity assay

The activity of the cytosolic and mitochondrial aconitase enzymes was determined by the use of the commercial kit (MAK051, Sigma-Aldrich, St Louis, MO), which is based on a colorimetric reaction, following provided instructions.

### Determination of Nrf2 activation

The binding activity of Nrf2 in nuclear extracts was quantified using an ELISA-based

TransAM kit (Active Motif, Carlsbad, CA, USA) following the manufacturer´s protocol. This experiment was completed with the quantification by Western blotting of the nuclear and cytosolic fractions of Nrf2 protein.

### Western blot analysis

Equivalent amounts of protein (20 μg from the adequate cellular extract) for each assay were separated on 10%-15% SDS-polyacrylamide gels and transferred onto PVDF membranes. After Western blotting, membranes were placed in blocking solution (5% milk powder in PBS-Tween buffer, pH 7.4) and blocked at room temperature for 90 min. Membranes were then washed gently and incubated overnight at 4°C with the appropriate dilutions of the primary antibodies in PBS with 0.1% BSA (caspase-3 1:2000, Bax 1:5000, Bcl-2 1:5000; cytochrome C: 1:5000; Nrf2 1:500; ß-actin 1:50000; TFIIB 1:500). The following day, the blots were washed in PBS-tween and incubated with the corresponding horseradish peroxidase-conjugated secondary antibody for 2 h at room temperature (anti-mouse and anti-rabbit antibodies 1:2000). Finally, membranes were washed again with PBS-Tween prior to revelation with ECL Prime detection kit (GE Healthcare, UK). The densitometric analysis of protein bands was carried out by using an Image Quant LAS500 analyzer (GE Healthcare) and the software ImageQuant 5.2. Protein expression in control cells was taken as 100% and results for the different treatments expressed as relative percentage. The β-actin was used as the housekeeping control for cytoplasmic proteins and the TATA-binding protein TFIIB as the loading control for nuclear proteins.

The cytosolic extracts were prepared from the total cellular pellets (collected after treatments) which were resuspended in an appropriate lysis solution (10 mM HEPES (pH 7.9), 1 mM DTT, 10 mM KCl, 5 mM NaF, 1 mM EDTA, 1 mM EGTA, 1 mM NaVO_4_, 10 mM Na_2_MoO_4_, 0.5 mM PMSF, 10 μg/ml leupeptin and 1 μg/ml pepstatin) and kept in ice for 15 min. Following incubation, cell lysis was specifically favored with 10% Nonidet P-40 and samples were centrifuged (13000 rpm, 30 s, 4°C) for collecting the supernatants that contained the purified cytosolic extracts. The resulting cellular pellets were used for the preparation of nuclear extracts. Therefore, they were initially resuspended in another lysis buffer (20 mM HEPES (pH 7.9), 1 mM DTT, 5 mM NaF, 1 mM EDTA, 1 mM EGTA, 0.4 mM NaCl, 20% glicerol, 1 mM NaVO_4_, 10 mM Na_2_MoO_4_, 0.5 mM PMSF, 10 μg/ml leupeptin and 1 μg/ml pepstatin) and then shaken on vortex for 30 min at 4°C. Once lysed, samples were finally centrifuged (15000 rpm, 5 min, 4°C) and nuclear extracts were obtained (supernatants).

### Blood brain barrier (BBB) permeability studies

#### Cell cultures

The human brain capillary endothelial hCMEC/D3 cells were used as an *in vitro* BBB model [35], obtained from Tebu-Bio (Barcelona, Spain). Cells between passage 25 and 35 were grown in with the EBM-2 medium (Lonza, Basel, Switzerland) supplemented with 5% FBS, 1% penicillin/streptomycin, 10 mM HEPES, bFGF, 1.4 μM hydrocortisone, 5 μg/ml ascorbic acid and chemically defined lipid concentrate (1/100). Cells were cultured in collagen pre-coated sterile petri dishes at 37°C, 5% CO_2_ and saturated humidity, and medium was changed every 2–3 days [36].

#### Permeability studies

For the transport experiments, the hCMEC/D3 cells were seeded in 12-well, 0.4 μm porosity PET transwell plates (Corning) in a density of 2×10^4^ cells/cm^2^ and allowed to grow the sufficient time to form a monolayer. In order to assure the structural integrity and the formation of the tight junctions in the monolayer, transendothelial electrical resistance (TEER) was periodically examined in the cells culture with an EVOM2 Epithelial Voltohmmeter and a STX2 electrode (World Precision Instruments, Sarasota, FL, USA). TEER was found to gradually increase during the cultures from low values of 9–10 Ω×cm^-2^ (at day 2) to values closer to 50 Ω×cm^-2^ after 12–14 days of culture. Additionally, the quality of the monolayer was assessed by the permeation study of a highly hydrophilic compound with low molecular weight such as Lucifer Yellow [37]; following transwell manufacturer´s protocol, a flux of LY lower to 2% indicated good integrity of the monolayer.

For transport assays (apical to basal direction), ginsenosides Rb1 and Rg1 were dissolved at the desired concentration in HBSS buffer with 10 mM HEPES (< 0.1% DMSO) and 300 μL were added to the upper chamber (mimicking the blood compartment), while 1000 μL of HBSS buffer were added to the lower chamber (mimicking the cerebral compartment). Plates were then incubated for 24 h with the test concentrations and, at the end of this period, 500 μL of the solution in the lower chamber were removed and samples were analyzed through HPLC for the quantification of the ginsenosides fractions that permeated (following the method described by Lau and coworkers [38]). The apparent permeability of each compound was calculated following the formula: P_app_ = (Flux x V_d_) / (t x A), where *Flux* is the fraction of the donated amount recovered in the receiver chamber, *V*_*d*_ is the volume in the donor chamber [cm^3^], *A* is the surface area of insert filter membrane [cm^2^] and *t* is the incubation time [sec].

#### Estimation of molecular properties

ALOGPS 2.1 program (http://www.vcclab.org) was used to estimate the physicochemical properties of ginsenosides Rb1 and Rg1 such as lipophilicity, represented by log P (partition coefficient n-octanol/water), and molecular weight. Similarly, topological polar surface area (TPSA), defined as the sum of surface contributions of polar atoms in a molecule (nitrogen, oxygen and hydrogen atoms), was determined using the Daylight Chemical program (http://www.daylight.com) [39]. The Molinspiration program (http://molinspiration.com/cgi-bin/properties) allowed the calculations of the number of hydrogen bond donors (HD) and acceptors (HA) and number of rotatable bonds.

### Statistics

All experiments were performed independently and in triplicate, and their results are expressed as mean values ± standard deviation (SD). Statistical significance was analyzed by one-way ANOVA followed by the Tukey´s test for multiple comparisons through the use of the Statgraphics Centurion XVI software. Results were considered significant at p < 0.05.

## Results and discussion

### ORAC assay

Compounds exerting antioxidant effects can directly interact with ROS; actually, the scavenging of free radicals and reactive species has been proposed as a mechanism by which the oxidative damage can be reduced in biological systems [40]. As a first approach to the antioxidant potential of ginsenosides, their capacity to scavenge peroxyl radical *in vitro* was evaluated by the chemical ORAC test. We observed that the chemoluminiscence induced by the peroxyl radical generation (initiated by AAPH thermal decomposition) decreased with the addition of increasing doses of ginsenosides. Such effect is probably due to the donation of a hydrogen atom to the unstable radicals, which is the fundament of the method. They presented different degree of activity (Table 1): the most active compounds were ginsenosides Rb1 and Rg1, while the ginsenosides Re and Rc exerted the lowest activity. Still, all ginsenosides showed interesting radical scavenging capacities, with higher ORAC values than other natural products previously studied [41].

**Table 1:** ORAC values obtained from the six ginsenosides screened for radical scavenging capacity.

Compound	ORAC value (μmol TE/mg)
Ginsenoside Rb1	1.55 ± 0.14
Ginsenoside Rb2	1.10 ± 0.10
Ginsenoside Rc	0.84 ± 0.12
Ginsenoside Rd	1.26 ± 0.13
Ginsenoside Re	0.74 ± 0.10
Ginsenoside Rg1	1.28 ± 0.12

### Effects of ginsenosides on cell viability and cytoprotection against rotenone-induced cytotoxicity

Firstly, we aimed at determining the range of concentrations of the different ginsenosides that do not compromise the viability of SH-SY5Y cells. SH-SY5Y cell line (derived from human neuroblastoma) is a common model of neuronal cells which is widely used for the *in vitro* examination of the effects of exogenous compounds on neuron viability and biological changes; they are considered as dopaminergic cells with high sensitivity and low variability [23].

The MTT assay, which measures the activity of mitochondrial dehydrogenases as an indicator of living cells, was used to test a vast range of concentration from 0.5 up to 50 μM for all six compounds. In general, we found that 24 h treatments with ginsenosides at concentrations higher than 10 μM exerted a remarkable cytotoxicity (data not shown), and the lower doses were chosen for testing their cytoprotective effects against rotenone. Concerning rotenone model, several doses were assayed in a 24 h treatment and we selected a model of toxicity in which there was a 40% of cell death for subsequent determination of cytoprotective actions by ginsenosides. Such effect corresponded to a concentration of 50 μM of rotenone, which was in agreement with previous studies [27].

**Fig 1:**
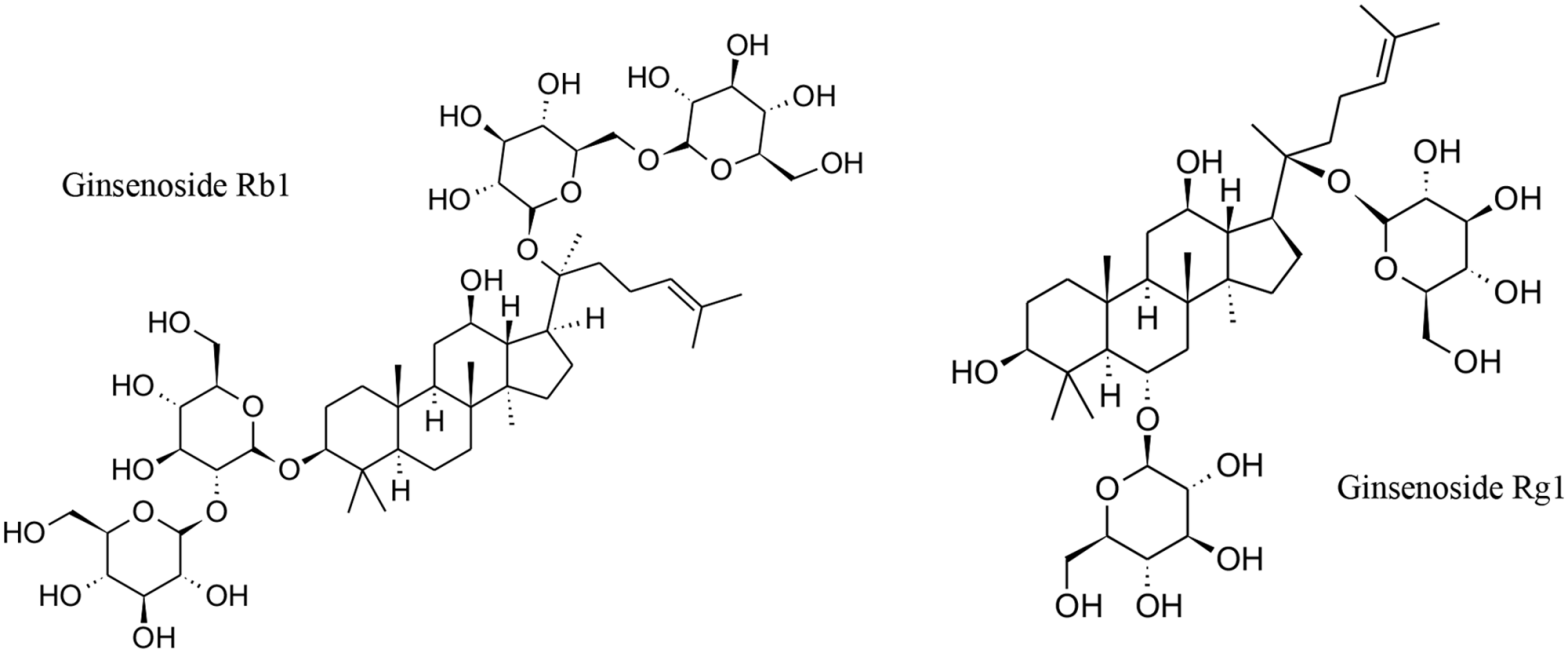
Chemical structures for the two ginsenosides under study.

**Fig 2:**
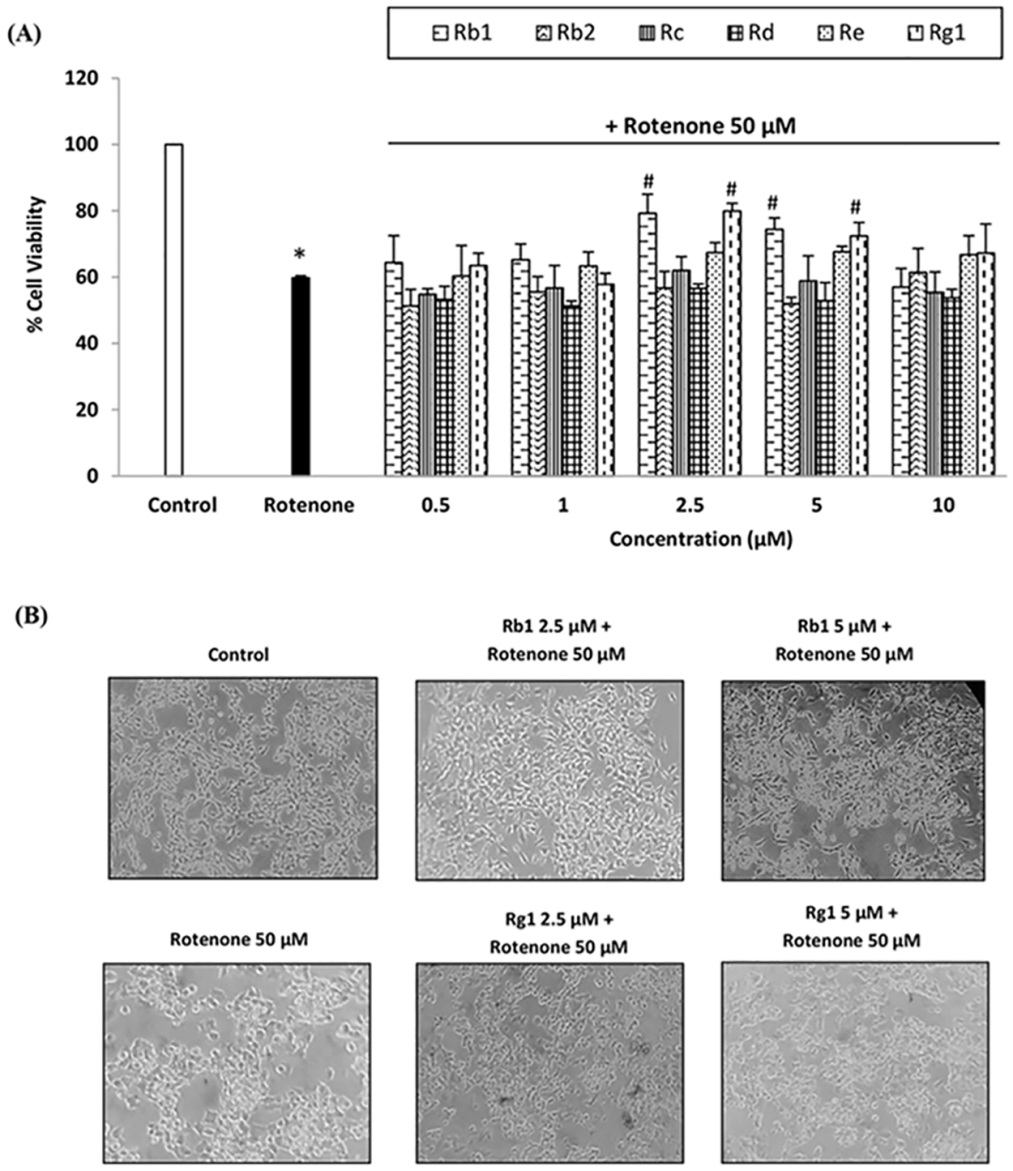
Cytoprotective effects of ginsenosides. (A) Cytoprotective effects of six ginsenosides in SH-SY5Y cell line against rotenone-mediated toxicity (range of concentrations: 0.5–10 μM), assessed by MTT reduction assay. Cells were pre-treated with ginsenosides for 24 h and then exposed to rotenone 50 μM for other 24 h. The viability of control cells was normalized to 100%. Means ± SD, * p < 0.05 Vs control, # p < 0.05 Vs rotenone. (B) Representative images of the morphological studies conducted on SH-SY5Y cell model with ginsenosides Rb1 and Rg1.

At this point, we evaluated the effect of 24 h pre-treatments with ginsenosides on the reduction in cell viability mediated by the established rotenone model. We found that the most effective compounds were ginsenosides Rb1 and Rg1 (Fig 1), both at the concentrations of 2.5 and 5 μM (see Fig 2A). They promoted cell survival with a significant increase of 15–20% in MTT reduction compared to cells only treated with rotenone. Therefore, these two compounds and optimal doses were chosen for the evaluation of their adaptive effect (and mechanism of action) in SH-SY5Y cells against the oxidative stress and mitochondrial dysfunction provoked by the exogenous administration of rotenone. Previous research on ginseng suggest that ginsenosides Rb1 and Rg1 are the most active and abundant metabolites in all species of *Panax* genus and have been used in similar concentrations for the evaluation of their pharmacological potential [42,43].

Moreover, the selected doses of ginsenosides demonstrated a protective capacity against the deleterious morphological changes observed after rotenone treatment, which mainly provoked a reduction in the size of SH-SY5Y cells and the loss of cell prolongations; cells also looked more rounded and started to detach from plate surface. Ginsenosides Rb1 and Rg1 partially preserved normal cell size and shape (Fig 2B).

### Ginsenosides Rb1 and Rg1 attenuate the rotenone-induced changes in oxidative stress markers

Rotenone has been shown to trigger a harmful oxidative stress situation in CNS-like cells, both in *in vivo* and *in vitro* models. It involves DNA damage, alterations in antioxidant contents (such as glutathione) and in proteins function and structure; it also increases membrane lipids peroxidation and intracellular ROS levels, among others consequences. In turn, all these abnormalities lead to cellular perturbation and subsequent degeneration [44,45]. Herein, we aimed at assessing the capacity of ginsenosides Rb1 and Rg1 to minimize such changes. Results are collected in Table 2.

**Table 2:** Effects of ginsenosides pretreatments (24 h) on the rotenone-induced alterations in oxidative stress markers.

Cell treatment	Ratio GSH/GSSG	Intracellular ROS production (% Vs control)	TBARS levels (% Vs control)	SOD activity (IU / mg protein)
Control	6.99 ± 1.20	100.00	100.00	18.01 ± 2.11
Rotenone 50 μM	1.40* ± 0.38	147.28* ± 14.65	182.11* ± 21.52	11.41* ± 1.40
Rb1 2.5 μM + Rot.	7.55^#^ ± 0.37	117.79^#^ ± 2.78	111.47^#^ ± 13.33	23.62^#^ ± 4.02
Rb1 5 μM + Rot.	6.86^#^ ± 1.09	109.00^#^ ± 2.33	143.70^#^ ± 11.35	22.75^#^ ± 1.51
Rg1 2.5 μM + Rot.	8.08^#^ ± 2.65	106.80^#^ ± 0.80	139.50^#^ ± 19.59	23.39^#^ ± 3.22
Rg1 5 μM + Rot.	5.00^#^ ± 1.64	128.88^#^ ± 8.37	143.50^#^ ± 16.51	22.28^#^ ± 1.88

Results are expressed as the mean value ± SD.

* p < 0.05 Vs control,

^#^ p < 0.05 Vs rotenone.

The major intracellular non-enzymatic antioxidant system in the brain cells is represented by glutathione [46]. The levels of its reduced active form and the oxidized one were quantified by a fluorimetric method using o-phthalaldehyde (OPT) as the fluorophore. We found that rotenone significantly diminished the content of GSH in comparison to control cells, while the contrary effect was found in GSSG levels; this resulted in a 4-fold reduction of the ratio GSH/GSSG. A pre-treatment with ginsenosides Rb1 and Rg1 significantly prevented the depletion of glutathione in SH-SY5Y cells, thus ameliorating the redox status.

The effect of ginsenosides and exogenous rotenone on the intracellular ROS levels were evaluated by measuring 2´,7´-dichlorofluorescein (DCF) fluorescence. We found that a treatment (24 h) with only the ginsenosides Rb1 and Rg1 did not provoke any significant change in ROS content (data not shown), what means that they do not induce oxidative stress by themselves. Data in Table 2 reflect, however, that a 24 h rotenone treatment remarkably enhanced the intracellular ROS levels (up to 150%), as previously revealed [47]. When pre-treated with ginsenosides, cells presented lower levels of intracellular ROS in comparison to rotenone-treated cells, implying that the cytoprotective effects of ginsenosides Rb1 and Rg1 at their optimal doses is in part mediated by the reduction of ROS-induced damage [48].

The oxidative peroxidation of lipids in cell membranes is considered to be a crucial mechanism of cellular damage caused by oxidative stress and is widely used as a biochemical marker in the pathophysiology of CNS neurodegenerative disorders [49]. Since an anti-lipid peroxidative effect was reported for ginsenosides Rb1 and Rg1 [50], we aimed to confirm such activity in the rotenone model in SH-SY5Y cells. Expressed as TBARS levels, there was almost a 2-fold increase in peroxidation products in rotenone-treated cells when compared to controls. However, a pretreatment with ginsenosides prior to rotenone exposure reduced such alteration. The ginsenoside Rb1 at the concentration of 2.5 μM displayed the most effective diminution of lipid peroxidation.

Anion superoxide (O_2_^·-^) is one of the most reactive radicals within the cell and worsens the oxidative stress-mediated cytotoxicity when it is over-generated (mainly in mitochondria). SOD enzyme decomposes the anion superoxide (O_2_^·-^) to O_2_ and H_2_O_2_, thus attenuating its deleterious effects; SOD comprises a cytosolic (SOD-1, Cu-Zn-dependent) and a mitochondrial isoform (SOD-2, Mn-dependent) and its activity is really important for the maintenance of redox homeostasis and cell survival. What is more, deficiencies in SOD have been linked to neurodegenerative disorders [51]. Exposure of SH-SY5Y cells to rotenone 50 μM significantly diminished their potential to remove the anion superoxide, as the SOD activity was reduced to almost half of normal value in control cells; such results are in line with similar findings *in vivo* [52]. Pretreatments with ginsenosides Rb1 and Rg1 also ameliorated the situation of rotenone-induced oxidative stress by enhancing SOD activity, reaching even higher levels than those found in control cells.

### Effects of ginsenosides in Nrf2 pathway

The above-mentioned effects of ginsenosides Rb1 and Rg1 on the SOD activity suggested that SOD expression could have been augmented in cells pre-treated with ginsenosides. Since the expression of SOD and other phase-II antioxidant enzymes is regulated by the transcription factor Nrf2, and in view of the positive results in oxidative stress markers (which imply an enhanced antioxidant potential in pre-treated cells), we hypothesized that the cytoprotective effect of the tested ginsenosides in the present model could involve the activation of Nrf2 signaling pathway; this is a commonly accepted approach to achieve chemoprevention against oxidative stress. Under normal circumstances, Nrf2 is in its inactive form (bound to Keap1) in the cytosol, but in the presence of an activator signal or compound (e.g. electrophilic drugs), Nrf2 is released from this union and translocates to nucleus. In turn, it activates the antioxidant response element (ARE) and the corresponding genes, thus enhancing the expression of cytoprotective proteins, such as those of the antioxidant enzymes [53].

**Fig 3:**
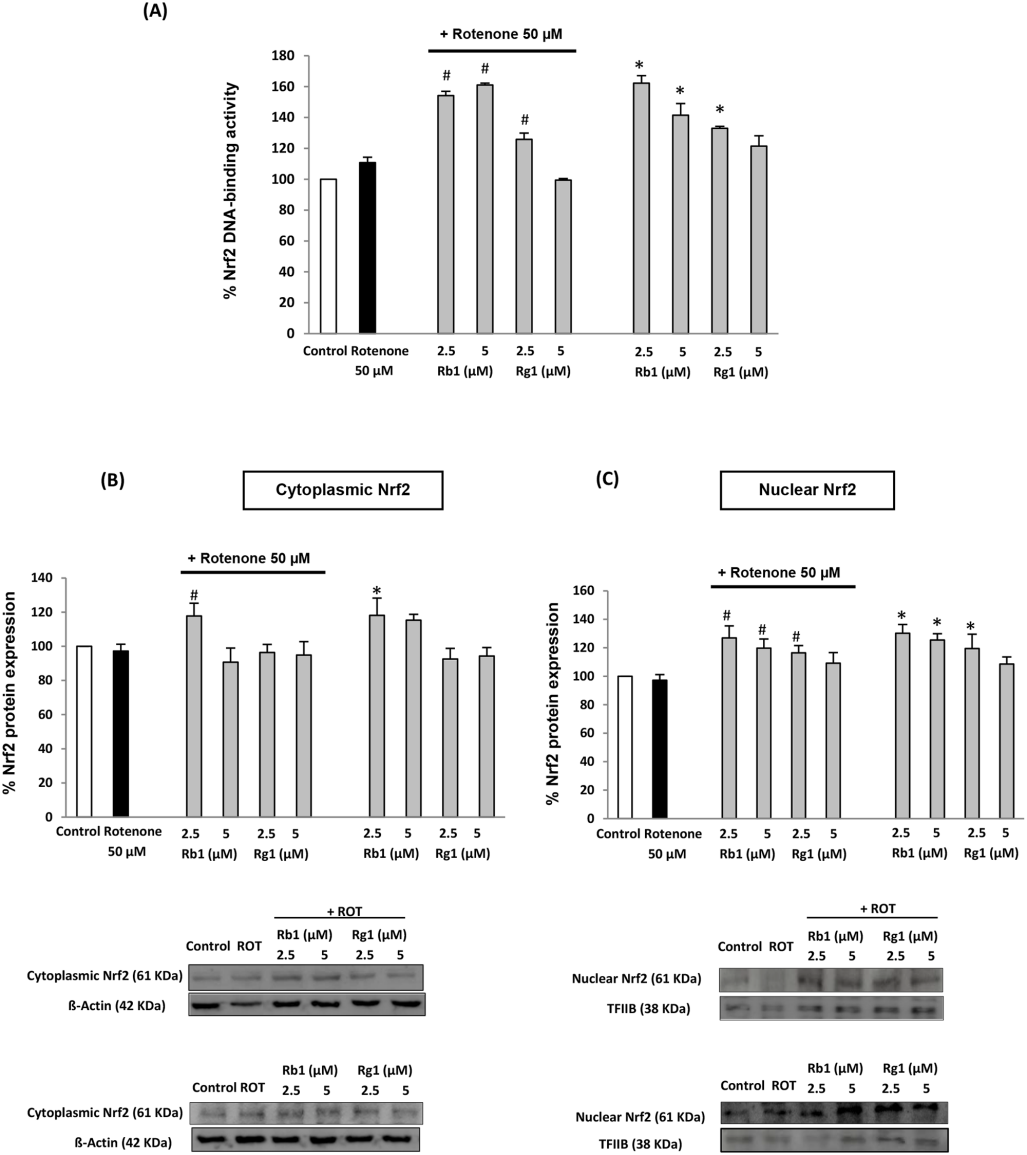
Involvement of Nrf2 signaling pathway in the cytoprotective actions of ginsenosides Rb1 and Rg1. (A) Degree of activation of Nrf2 factor in nuclear extracts of SH-SY5Y cells. (B) Effects of ginsenosides on Nrf2 protein levels in cytoplasm and (C) in nucleus; representative blots from purified cytoplasmic and nuclear fractions. Cells were pre-treated with ginsenosides for 24 h with/without later exposure to rotenone 50 μM for other 24 h. Data are expressed in percentage relative to untreated control cells. Means ± SD, * p < 0.05 Vs control, # p < 0.05 Vs rotenone-treated cells.

Through an ELISA-based assay (TransAM kit), we evaluated the level of activation of Nrf2 in the nuclear extracts of SH-SY5Y cells under different treatments. This method evaluates the capacity of nuclear Nrf2 protein to bind DNA, which is the crucial requirement for this factor to activate and modulate gene transcription. The treatment of cells with only ginsenosides Rb1 (2.5 and 5 μM) and Rg1 (2.5 μM) provoked a significant activation of Nrf2 factor in comparison to untreated cells. On the other hand, no significant change was found in the rotenone-treated group, in which the DNA binding activity of Nrf2 was similar to that of control cells. However, if cells were pretreated with ginsenosides before rotenone exposure, again enhanced DNA binding activity was achieved (statistically significant in comparison to rotenone group), what can be attributed to the initial activation triggered by ginsenosides (Fig 3A).

Finally, in order to better understand and confirm the involvement of this signaling pathway in the neuroprotective effects of ginsenosides, we further assessed their effects on the Nrf2 protein expression levels. Since the translocation of Nrf2 factor from cytoplasm to nucleus is a key pre-requisite for its activity, we quantified by Western blot the levels of Nrf2 protein in the cytoplasmic (Fig 3B) and nuclear (Fig 3C) fractions. Results show that cells only exposed to rotenone did not presented a statistically significant change in the content of nuclear Nrf2 (which was only slightly reduced) when compared to control cells. However, when pre-treated with Rb1 and Rg1 (24 h), nuclear levels of Nrf2 protein were remarkably increased in comparison to rotenone-treated cells, especially in the case of Rb1 (2.5 and 5 μM). Such results confirm the effect evidenced by the only treatments with ginsenosides, which were also capable to enhance the levels of nuclear Nrf2 (Fig 3C). Our data point to the induction of Nrf2 protein translocation, since the increase in nuclear levels is statistically significant without altering much basal cytoplasmic Nrf2 content (Fig 3B). Thus, one could suggest that ginsenosides Rb1 and Rg1 are capable to promote translocation and stabilize Nrf2 transcription factor in the appropriate cellular compartment (nucleus) where it should display its regulatory activity of gene transcription.

Together with the enhanced DNA-binding activity, our results are in agreement with other studies that reported Nrf2-activator activity for the ginsenosides Rb1 and Rg1 in similar models [48,54] and point to the involvement of Nrf2 signaling pathway in the cytoprotective activity of ginsenosides. A manipulation of such pathway is required for a further characterization of its involvement.

### Effects of ginsenosides in mitochondrial dysfunction

Rotenone is a pesticide and a complex I inhibitor that causes nigrostriatal degeneration similar to Parkinson´s disease pathology in a chronic systemic *in vivo* rodent model [55]. Inhibition of complex I provokes the disruption of mitochondrial electron transport chain and, in turn, leads to alteration of energy metabolism and mitochondrial dysfunction, which is a key pathogenic feature in many neurodegenerative diseases. In addition, there is a close relationship between mitochondrial failure and oxidative stress, since mitochondria are the major cellular source of ROS and the most sensitive primary target for oxidative damage in neuronal cells at the same time [56].

One of the first changes in mitochondrial biodynamics induced by oxidative cytotoxicity is the mitochondrial membrane potential dissipation, which is mainly due to outer membrane rupture. Therefore, through a fluorimetric method involving the use of the dye TMRM, we assessed the capacity of ginsenosides Rb1 and Rg1 to prevent the loss of MMP (of around 40%) after rotenone exposure in SH-SY5Y cells. We demonstrated that cells only treated with ginsenosides presented levels of MMP similar to control (data not shown). Interestingly, it was also demonstrated that rotenone-induced MMP loss was significantly blocked by the pretreatment with ginsenosides Rb1 and Rg1, what implies that mitochondria maintained normal function and structure. Our results are in accordance to other previous studies that suggested a protective effect of ginsenosides on the MMP loss in different models [57,58]. In particular, Rb1 was more effective than Rg1 at preventing cells from MMP loss (Table 3).

**Table 3:** Effects of ginsenosides on the parameters used as bioindicators for rotenone-induced mitochondrial dysfunction.

Cell treatment	MMP (ΔΨm) (% Vs control)	Mitochondrial calcium uptake (% Vs Control)	Cytosolic calcium (nM)		Mitochondrial aconitase activity (IU / mg protein)	Cytosolic aconitase activity (IU / mg protein)
**Control**	100	100		69.48 ± 5.67	158.10 ± 8.52	107.74 ± 22.30
**Rotenone 50 μM**	58.15* ± 7.1	152.36* ± 5.35		256.45* ± 26.19	83.52* ± 3.24	68.97* ± 7.92
**Rb1 2.5 μM + Rot**.	88.78^#^ ± 13.27	111.05^#^ ± 4.04		120.98^#^ ± 23.15	152.08^#^ ± 14.56	132.39^#^ ± 16.15
**Rb1 5 μM + Rot**.	92.16^#^ ± 10.62	125.21^#^ ± 2.61		190.31^#^ ± 13.13	104.00^#^ ± 8.46	86.00 ± 10.58
**Rg1 2.5 μM + Rot**.	85.58^#^ ± 9.67	117.81^#^ ± 5.51		154.22^#^ ± 24.86	147.71^#^ ± 7.77	105.08^#^ ± 5.49
**Rg1 5 μM + Rot**.	75.19^#^ ± 2.56	132.35^#^ ± 3.35		225.15 ± 30.57	81.25 ± 6.02	88.55 ± 3.54

Cells were pretreated (24 h) with ginsenosides and then exposed to rotenone (24h). Results are expressed as the mean value ± SD.

* p < 0.05 Vs control,

^#^ p < 0.05 Vs rotenone.

The homeostasis of calcium ions is very important for maintaining mitochondrial viability. Calcium overloads in cytosol and mitochondria occur in neurons of neurodegenerative disease patients, and lead to aberrant events within these organelles, including the over-generation of ROS, mPTP opening and the dissociation and release of cytochrome C [59]. By using the fluorescent probe Rhod-2, we evaluated the mitochondrial calcium uptake and found that it was enhanced more than 0.5-fold in rotenone-treated cells compared to controls; an altered integrity of mitochondrial membrane and the subsequent higher permeability may be the cause of such result. When SH-SY5Y cells were pre-treated for 24 h with ginsenosides Rb1 and Rg1, prior to rotenone treatment, a decrease in mitochondrial calcium accumulation was observed. This effect was particularly marked for ginsenoside Rb1 (2.5 μM) pretreatment; it caused a reduction in mitochondrial calcium levels, which were similar to those in control cells (Table 3).

On the other hand, cytosolic calcium levels in SH-SY5Y cells suffered an increase higher than 3-fold after rotenone exposure (256.45 nM vs 69.48 nM in control cells). This finding might be explained by the fact that cellular oxidative stress affects normal calcium storages, such as the endoplasmic reticulum, and provokes the release of calcium to cytosol, thus affecting its homeostasis. Moreover, calcium imbalance aggravates the mitochondrial dysfunction [60]. Conversely, we demonstrated that pretreatments with ginsenosides significantly reduced calcium overload in the cytosol (with the exception of Rg1 5 μM). The effects of ginsenosides in calcium homeostasis could be expected by the results reported in previous works [61,62]. None of the assayed ginsenosides, at the concentrations and incubation times used, produced changes in either cytosolic or mitochondrial calcium levels by themselves.

Aconitase is an iron-sulfur containing enzyme that catalyzes the isomerization of citrate to isocitrate, and participates in the tricarboxylic cycle. In human tissues, it is present in cytosolic and mitochondrial (two isoforms) and is reversibly inactivated by oxidative stress (e.g. by the attack of anion superoxide); with this respect, it is revealed as an appropriate biomarker for oxidative damage, mainly in mitochondria. Herein, we showed that aconitase activity was reduced in approx. 50% in cells treated with only rotenone. The inactivation of aconitase is an index of free radical production and mitochondrial dysfunction provoked by rotenone [63]. Conversely, when cells were pretreated with ginsenosides Rb1 and Rg1 (2.5 μM) before rotenone exposure, the activity of the enzyme in both mitochondrial and cytosolic compartments was almost completely recovered to normal levels (Table 3), thus ameliorating the energy metabolism in the oxidative stress model.

### Ginsenosides Rb1 and Rg1 attenuate mitochondria-mediated apoptosis

According to the results presented above, rotenone generated a significant percentage of cell death. Since rotenone is known to be an efficient pro-apoptotic agent in SH-SY5Y cells [64], we evaluated several parameters related to this cell death pathway, which is very common in the neuronal death present in age-related neurodegenerative disorders. Apoptosis is also closely linked to oxidative stress and mitochondrial dysfunction, and actually ROS can stimulate the intrinsic mitochondrial apoptotic cascade leading to mitochondrial DNA damage and release to cytosol of pro-apoptotic proteins such as Bax and cytochrome C [65].

**Fig 4:**
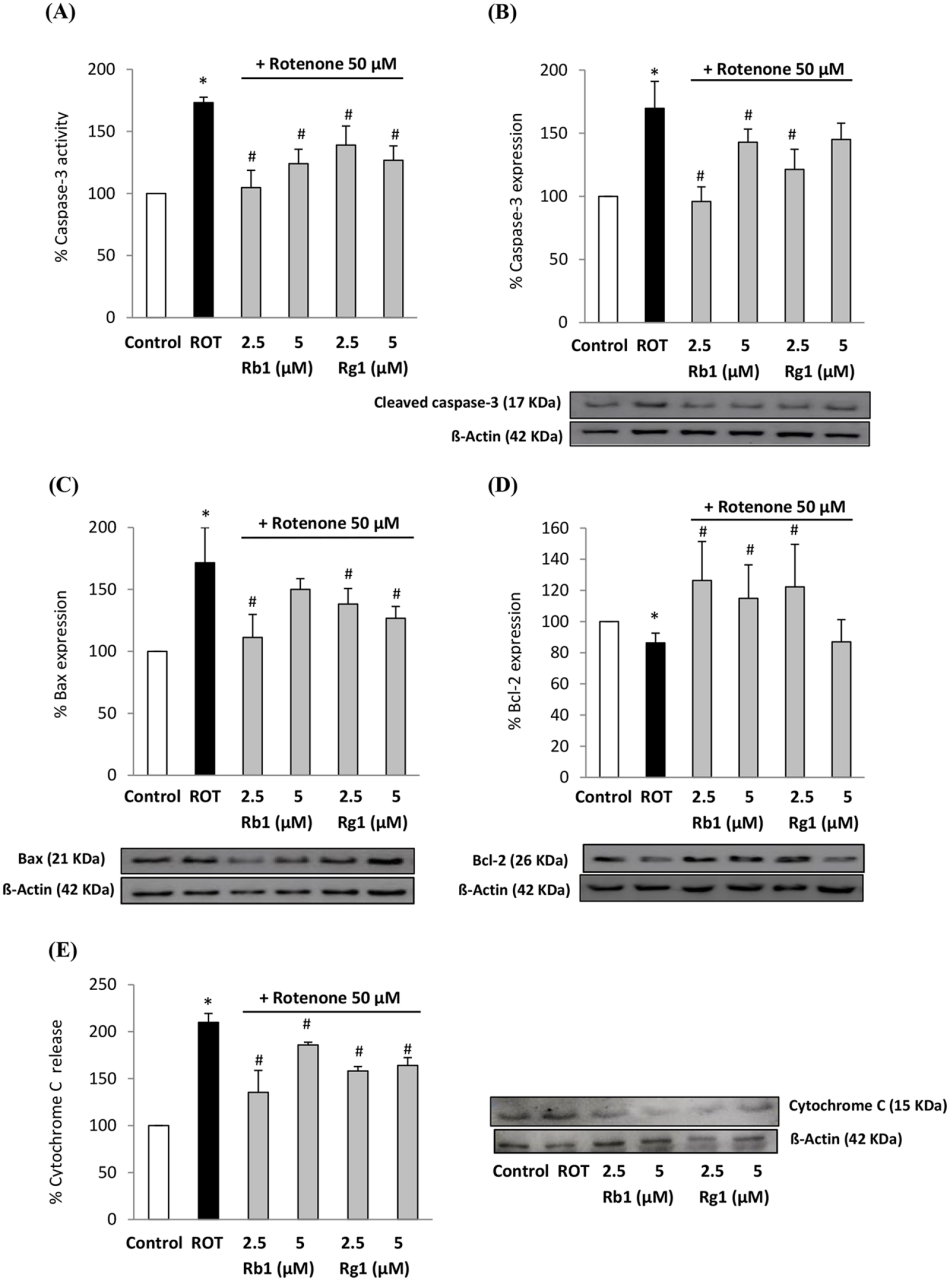
Effects of ginsenosides Rb1 and Rg1 on apoptosis-related factors. (A) Caspase-3 activity and (B) protein expression, (C) Bax expression, (D) Bcl-2 expression and (D) cytosolic cytochrome C. Cells were pre-treated with ginsenosides (optimal doses, 24 h) and then exposed to rotenone (50 μM, 24 h). Data are expressed in percentage relative to control cells (100% of enzyme activity and expression). Means ± SD, * p < 0.05 Vs control, # p < 0.05 Vs H_2_O_2_.

Firstly, we assessed through a fluorimetric method the activity of the effector enzyme caspase-3, which triggers DNA fragmentation when activated and is used as a hallmark of apoptosis. It was evidenced that the activity of the enzyme was enhanced more than 70% in SH-SY5Y cells treated with rotenone, in comparison to control cells. Ginsenosides Rb1 and Rg1 pretreatments exerted a significant inhibitory action of such over-activation, thus reducing the rotenone-induced apoptotic cell death (Fig 4A). Further, an enhanced apoptosis is often mediated by an increase in pro-apoptotic proteins expression. We therefore used Western blotting for the confirmation of such results. Consequently, we found that the overexpression in cleaved caspase-3 mediated by rotenone exposure was attenuated by a pretreatment with ginsenosides Rb1 and Rg1 (Fig 4B).

Inmunoblot was also used for the quantification of the expression of Bax and Bcl-2 in total extracts (Fig 4C–4D), which are representative pro-apoptotic and anti-apoptotic factors, respectively. Both proteins play important roles in regulating cell death and survival [66]. As compared with the controls, in rotenone-treated neuronal cells we found a significant increase in Bax expression, while the effect in Bcl-2 expression was the contrary. In contrast, when cells were pre-treated with the optimal doses of ginsenosides, the effects provoked by rotenone were reversed. What is more, the expression of Bcl-2 protein was enhanced over the levels found in control cells. The treatments with 2.5 μM of ginsenosides Rb1 and Rg1 were the most effective in favoring the anti-apoptotic status, what is in line with the cytoprotective effects showed in Fig 2.

Cytosolic content of cytochrome C (Cyt C) is another marker for apoptotic cell death. The release of Cyt C from mitochondria to cytosol, due to decreased mitochondrial function and membrane disruption, leads to a series of signaling cascades culminating in apoptosis activation in multiple cell types. Our results on the cytosolic extracts of SH-SY5Y cells showed that rotenone treatment produced a 2-fold increase in Cyt C release from mitochondria (Fig 4E). Conversely, when cells were pre-treated with ginsenosides Rb1 and Rg1, the rotenone-induced release of Cyt C to cytosol was significantly reduced. These results are in accordance with those obtained for Bcl-2, since this anti-apoptotic protein located in the mitochondrial outer membrane inhibits the release of Cyt C [67]. The anti-apoptotic effect demonstrated for ginsenosides Rb1 and Rg1 in our study confirms the hints extracted from previous reports [58,68].

We showed that ginsenosides Rb1 and Rg1 display mitochondrial protective and anti-apoptotic effects at lower concentrations than those used in previous studies. For instance, Leung and coworkers [69] reported the effectiveness of ginsenoside Rg1 as a cytoprotective agent in a similar rotenone-based model in primary neurons. However, when used in co-treatment with rotenone, the anti-apoptotic effect of ginsenoside Rg1 was evidenced at a concentration of 50 μM. Therefore, our results imply that the pre-treatment with lower concentrations (2.5 and 5 μM) of the ginsenosides Rb1 and Rg1 enhance the antioxidant potential within the cells, thus preventing from the cytotoxic and apoptotic effects of rotenone.

### Estimation of the capacity of ginsenosides to cross the BBB

The neuroprotective potential demonstrated so far for ginsenosides Rb1 and Rg1 suggests that these two compounds may be potential candidates for the therapy of neurodegenerative disorders. A crucial requirement for neuroprotective agents (that has limited their efficacy in many cases) is their capacity to cross the BBB and exert their effects in the CNS. In this work, we approached to such issue and evaluated for the first time their capability to permeate across an *in vitro* model of BBB (the hCMEC/D3 cell line).

By using a HPLC method [38] and the use of two respective standard curves for ginsenosides Rb1 and Rg1, we aimed at quantifying their content in the basal chamber and to determine the flux of both ginsenosides through the monolayer that mimics BBB properties. Among those published, such HPLC method is one of the most sensitive and precise methods for the detection of ginsenosides. However, we could only quantify the concentration of ginsenosides Rb1 when the treatment was done at 5 μM. This means that lower doses of Rb1 and the ginsenoside Rg1 at both concentrations tested (2.5 and 5 μM) could not permeate through the hCMEC/D3 monolayer in a quantity enough to be detected. Rb1 presented an apparent permeability (P_app_) of 2.23x10^-8^ cm/s (Table 4), which is a low value, as previous studies established that P_app_ values greater than 3 × 10^−6^ cm/s suppose efficient permeation through the BBB [70].

**Table 4:** Estimation of the molecular properties involved in the capacity of a compound to achieve passive diffusion through the BBB.

Compounds	Rb1	Rg1
Molecular Formula	C_54_H_92_O_23_	C_42_H_72_O_14_
Molecular Weight (g/mol)	1109.29	801.01
Log P_oct_	0.24	1.00
Log Poct / MW	0.0002	0.0012
TPSA	377.29	239.22
HA (O+N)	23	14
HD (OH + NH)	15	10
Rotatable bonds	16	10
**P**_**app**_ **(cm/s)**	**2.23 x 10**^**−8**^	-

Furthermore, other authors proposed several rules for estimating the capacity of a compound to achieve passive diffusion through BBB. Among the requirements suggested, one may highlight the followings: molecular weight ≤ 450 Da, the sum of the nitrogen (N) and the oxygen (O) atoms in the molecule ≤ 5, the value of log P–(N + O) > 0 and TPSA value ≤ 90 Å [71,72]. None of these premises is satisfied by any of the two ginsenosides studied (see Table 4), that are big molecules with a large number of polar atoms and low lipophilicity.

Although some previous reports on the neuroprotective activity of these compounds imply their capacity to cross BBB [19], no study has clearly assessed so far their capacity to permeate towards the brain. The low capacity exhibited by ginsenosides Rb1 and Rg1 to permeate through BBB (under the experimental conditions used here) would mean that higher doses should be administered in order to reach effective concentrations in the brain or, instead, other possibilities such as the recent techniques of nanoencapsulation should be considered. We believe that further research is needed to better understand the capacity of ginsenosides to cross BBB; for instance, mechanisms of active transport could be investigated as well as *in vivo* models should be recommended, taking into consideration that many factors affect the bioavailability of drugs in animals.

## Conclusions

In the current report, we demonstrated for the first time the adaptogenic effect of ginsenosides Rb1 and Rg1 against rotenone-induced neurotoxicity in a model of neuronal cells. It was evidenced that these ginsenosides act at different levels of the neuronal degenerative process. Pre-treatments with ginsenosides Rb1 and Rg1 attenuated rotenone-induced oxidative stress and mitochondrial dysfunction, thus resulting in a reduced apoptotic cell death. The most promising effects in our SH-SY5Y model were found for ginsenoside Rb1 at the concentration of 2.5 μM. Such actions may be mediated, at least in part, by the activation of cytoprotective Nrf2 signaling pathway and a mitochondrial-targeted protective action.

The slight differences found in the pharmacological activities of ginsenosides Rb1 and Rg1 may be attributed to the differences in their chemical structures (Rb1 presents four sugars and Rg1 only two). The chemical structure also determine their capacity to permeate across the BBB by passive diffusion, which was found to be low in general terms; still, ginsenoside Rb1 showed better permeation than Rg1 in the *in vitro* BBB model.

Taken together, our results contribute to validate the ethnopharmacological use of ginseng preparations for cognitive-enhancing purposes in age-related neurodegenerative disorders. They provide basis to encourage further research on the real potential of ginsenosides Rb1 and Rg1 as valuable tools in the therapy of those diseases. Thus, the pharmacological potency of both compounds and their combinations should be confirmed in other *in vitro* and *in vivo* models.
